# Construction and validation of an instrument to assess the university dropout intention formation process UDIFP-29

**DOI:** 10.1371/journal.pone.0349293

**Published:** 2026-06-17

**Authors:** Yaranay López Angulo, Karla Muñoz-Inostroza, Fabiola Sáez-Delgado, Javier Mella-Norambuena, Carlos Patricio Zalaquett

**Affiliations:** 1 Departamento de Psicología, Facultad de Ciencias Sociales, Universidad de Concepción, Concepción, Chile; 2 THRIVE4ALL, Research and Innovation Group in Socioemotional Learning, Well-Being and Mental Health to Foster Thriving, Universidad Católica de la Santísima Concepción, Concepción, Chile; 3 Universidad Técnica Federico Santa María, Departamento de Ciencias, Concepción, Chile; 4 The Pennsylvania State University, University Park, State College, Pennsylvania, United States of America; University of Lahore - Raiwind Road Campus: The University of Lahore, PAKISTAN

## Abstract

A significant body of research supports the importance of addressing university students’ dropout intention, the process through which university students form the intention to dropout from their studies. However, available measurement tools failed to account for how dropout intention is formed over time. This research aimed to fill that gap by developing and validating a multidimensional self-report instrument to assess how the intention to dropout from the university is formed. Three studies were designed for this purpose. Study 1 used focus groups to explore how the intention to dropout emerges in freshman students enrolled at a university, with the purpose of developing a theoretical model to explain how the intention to dropout emerges. Study 2 focused on the content validation of the preliminary version of the instrument, which was carried out by expert judgment. Study 3 examined the construct validity of the instrument using exploratory and confirmatory factor analysis and analyzing its convergent and divergent validity. The results showed a model with good adjustment and adequate internal consistency indexes. In general, the instrument has adequate psychometric properties, proving to be reliable and valid for measuring the process of forming early university dropout intention. Use of this instrument will allow the design of timely strategies and interventions to reduce dissatisfaction, dropout ideation and dropout.

## Introduction

Dropout rates in higher education continue to be a global issue. The Organization for Economic Cooperation and Development OECD [1] reports an average worldwide dropout rate of 30%. Latin American and the Caribbean rates are even more concerning, with a reported 54% [2]. The United States, dropout rates vary with 24.1% of first-year students dropping out at their first attempt of university insertion and 40% dropping out at some point of their academic career [3]. Chile’s rates rise significantly above 17% [4]. These rates reflect the complexity and magnitude presented by university dropout globally.

University dropout has negative consequences at multiple levels: microsystemic (person, family, friends, habits, among others), mesosystemic (higher education institutions), exosystemic (educational policies and financial resources allocated to higher education) [5], and macrosystemic (social, global development, and labor market). These negative consequences increase risks of unemployment, low income, and mental health issues [6], and negatively affects university quality indices. Thus, the effects of dropout are far-reaching.

### Empirical evidence on factors influencing early university dropout

University dropout is a complex and multi-determined phenomenon that has been extensively studied from various methodological approaches. A meta-review on the subject identifies five broad categories of associated factors: individual, academic, institutional, economic, and cultural [7–12]. Among individual variables, affective-motivational factors are particularly salient, such as vocational choice, satisfaction with the degree program, and personal expectations [13,14]. Psychological variables such as procrastination, self-efficacy, academic commitment, and maladaptive emotions like pessimism or low self-esteem also play a significant role [15]. Academic factors such as performance, absenteeism, and grades are similarly important [16], alongside socioeconomic variables and previous school records, including Higher Education Entrance Exam and secondary school grades in the case of Chile [17]. Likewise, the role of contextual variables such as sense of belonging, teacher support, social relationships, and social and family satisfaction has been documented [18,19]. Integrative models, such as those proposed by Tuero et al. [20] and López-Angulo et al. [19], suggest that the intention to dropout is shaped by a dynamic interaction of personal, academic, and contextual factors.

In summary, early university dropout is a complex phenomenon influenced by a combination of factors, with the first year being a particularly critical period for student adaptation [19], as it is the stage in which the highest dropout rates are recorded. In this framework, satisfaction emerges as a key determinant in the initial adaptation process to university, decisively influencing the perception of adequacy, adjustment, or maladjustment, and in many cases triggering the early idea of dropping out. Empirical evidence suggests that this adaptation is heavily influenced by three key areas of satisfaction: career satisfaction, performance satisfaction, and social satisfaction. Career satisfaction seems to increase retention, while career dissatisfaction has consistently been identified as a primary determinant of dropout intention [21]. The congruence between a student’s interests, abilities, and their academic program is a critical protective factor against this dissatisfaction. A lack of fit with the chosen courses is often linked to feelings of disconnect [ 22,23] and can be exacerbated by factors such as procrastination, which leads to a perceived mismatch with the career path [24,25]. In fact, students’ interest in and expectations for their academic programs, along with their own academic performance, are considered to be among the main causes of university dropout [26]. Therefore, a strong sense of career fit and alignment is a fundamental element for university retention.

Beyond career alignment, a student’s perception of their own academic performance is crucial. Performance satisfaction is closely tied to the effective use of self-regulated learning strategies [27]. Students who fail to implement these strategies are more likely to experience academic exhaustion and a sense of disconnection from their studies [ 23,24], which in turn increases their likelihood of dropout [27]. Conversely, a sense of academic competence and an ability to manage one’s own learning process significantly contribute to university retention and success [27].

The third key factor is social satisfaction. A student’s ability to integrate into the university’s social fabric is as important as their academic integration [5]. Having a supportive network of friends is a well-established protective factor against dropout intention [19,28,29] and strong social connections are vital for academic success and overall well-being [19,30,31]. This sense of belonging and community directly contributes to a more positive and satisfying university experience.

Thus, an individual’s successful adaptation to the academic system is not only a matter of academic ability but also depends on their satisfaction with their career choice, their perceived academic performance, and their social integration.

### Concept of dropout, intention of dropout, and the process of forming dropout intention

Conceptually, dropout is considered as the withdrawal from the study program before obtaining the degree [32]; it happens when the students voluntarily cease their academic activities in the institution to which they belong for three consecutive periods [33]. University dropout can be characterized as a gradual process of disengagement (action crisis) from the academic goal of obtaining a university degree [34]. This process seems to be preceded by intentions to dropout, that some researchers considered by as predictors of actual dropout [35].

Dropout intention involves awareness and intention to change career or dropping out of the university [36,37]. This intention refers to the student’s thoughts related to ceasing their permanence in the educational program leading to a higher education degree, before achieving the degree [38]. It is also understood as part of a decision-making process developed during the early stages of the university experience, which is characterized by being dynamic and convergent with multiple factors [39]. Although the concepts of dropout and dropout intentions are now well established, little progress has been made in understanding how these intentions develop. Moreover, the factors contributing to the formation of dropout intentions have not been fully examined in the extant literature.

### Theoretical models of dropout

There are at least three main theoretical models for explaining dropout rates. The first corresponds to general psychological theories, focusing on psychological factors to understand and explain the phenomenon of university dropout. These included the Theory of Planned Behavior [40], which posits that a person’s intention to perform a specific behavior is the most immediate predictor of action and is influenced by three components: attitudes toward the behavior, subjective norms, and perceived control of the behavior. The Incentive Disengagement Theory [41] proposes a four-phase cycle in response to goal pursuit and subsequent dropout: vigor (or motivation), aggression, depression, and recovery. The Decision-Making Model [42], includes three phases: (1) pre-selection, where the decision situation is identified by generating options and seeking relevant information; (2) selection, where possible consequences are evaluated and a decision is made; and (3) post-selection, where the decision is executed and feedback is received. Finally, the Rubicon Action Phase Model [43] presents four phases that stimulate different mental states leading to the successful completion of the tasks that each phase requires. Phase 1 (pre-decision) is characterized by open-mindedness. During this phase, one assesses the feasibility of certain desires and the desirability of their outcomes, a process that culminates with the distinction of a specific goal. In phase 2 (pre-action) the individual considers the best way to pursue that goal and establishes concrete strategies to achieve it. In phase 3 (action phase), attention is focused on maintaining the course of action. Finally, in phase 4 (post-action) an objective evaluation of the outcome of that action is conducted.

The second set of theoretical models focus mostly on psychological, economic, sociological, and organizational factors, as well as the interaction between the student and the educational institution, which may influence the decision to dropout. Spady’s Model [44] adopts a sociological approach that emphasizes the student’s integration into the university environment and highlight is the impact of external factors and family background on dropout. Similarly, Tinto’s Model [32] uses a multidimensional perspective that considers social, psychological, economic, and organizational elements. They examine how individual characteristics, and external factors affect the student’s decision to continue or dropout from the university. This model emphasizes the importance of social and academic integration. In contrast, Bean’s Organizational Model [45] emphasizes factors linked to the student experience, such as commitment to the institution, satisfaction with the university experience, participation in extracurricular activities, and external conditions as influencing the decision to dropout. Likewise, Pascarella and Terenzin model [46] shows that university persistence and dropout depend on the interaction of individual, social, and institutional factors, highlighting the importance of academic and social integration on campus. Finally, Ethington’s Psychological Model [47] emphasizes the importance of a student’s values and expectations of success, academic self-concept, perception of difficulty of studies, and aspirations. These factors may be influenced by the student’s previous academic performance and family background.

The third theoretical explanation for explaining dropout intention offers two models: the Student Dropout Intentions Process Model [48], which describes a sequential and cognitive framework for understanding how decision-making develops. The process begins with a *perception of non-fit* between the students and their studies overall or the specific program content and context. This perceptual mismatch can lead to prolonged *thoughts of quitting/changing* the university program. The process continues with a *deliberation phase*, where values and expectations are evaluated, followed by an *information search phase* to explore alternatives. The process, which is related to decision-making and planning of concrete actions, may culminate in the *final decision to dropout*. On the other hand, Mashburn [36] proposes a psychological model in which satisfaction influences students’ awareness of dropping out (thoughts and intentions), which in turn directly affects dropout behavior. In this model, the student dropout awareness, which includes thoughts of dropping out, search intentions, and dropout intentions, mediates the relationship between students’ satisfaction and dropout.

While each of the presented models contributes significantly to the understanding of dropout, they do not address the dropout intention formation process. It is important to explore how the intention to dropout forms in students, as it seems to be a precursor or predictor of the final decision to dropout. Therefore, this research proposed and developed a specific theoretical model and its corresponding instrument to measure intention to dropout.

### Instruments for measuring university dropout intention and knowledge gaps

Several instruments have been developed to assess university dropout and dropout intention, typically grounded in the theoretical perspectives described above. However, a critical review of these tools reveals significant conceptual, methodological, and contextual limitations. Most existing instruments rely on ad hoc item construction, often combining items from unrelated scales or developed without a strong theoretical foundation. As a result, they frequently lack robust psychometric validation, limiting the reliability and generalizability of their findings [9]. A systematic review [49] identified six main instruments used in the field: (1) “Early University Dropout Intentions Questionnaire” (EUDIQ-R) [50]; (2) “Screening Instrument for Students at-Risk of Dropping out from Higher Education” [51]; (3) “Questionnaire for the Analysis of University Student Desertion” (CADESUN, Spanish acronyms) [52]; (4) “College Persistence Questionnaire” (CPQ) [53]; (5) “WWH-Dropout Scale” [54]; and (6) “Scales to Assess Student Dropout Intentions” [48].

While these instruments provide some valuable information, they present several common shortcomings: (a) Focus on predictors rather than process: Five of the six instruments primarily identify causal variables (e.g., dissatisfaction, low motivation, institutional barriers) without modeling dropout intention as a dynamic or sequential process [48]. Only one instrument [48] aimed to capture dropout as a cognitive process, but still focused on the decision-making stages rather than the underlying psychological experience of disengagement. (b) Conceptual ambiguity: Many instruments use the terms “dropout” and “dropout intention” interchangeably, despite evidence that these are distinct constructs requiring separate operationalization and measurement [50,51,53,55]. (c) Weak alignment with theoretical models: Although most tools claim to be theory-based, they often fail to reflect those models in the structure or dimensions of the scale. No existing instrument fully and systematically operationalizes a theoretical model of dropout intention formation. (d) Limited exploration of psychological mechanisms: Critical mechanisms, such as academic disengagement, career dissatisfaction, or self-regulation failure, are not sufficiently addressed. This leaves a gap in our understanding of how dropout intention forms and evolves over time. (e) Insufficient psychometric validation: Several instruments show limited evidence of construct validity, internal consistency, or cross-cultural reliability. Very few are designed to measure intention formation during the early stages of university life-when dropout risk is highest [21]. (f) Additionally, most instruments have been developed in North America or Europe, with little adaptation to Latin American cultural, institutional, or linguistic contexts. Furthermore, even validated Spanish-language tools often fail to reflect the structural and contextual characteristics of the Chilean educational landscape.

### Closing the Gap: A Justification for the UDIFP-29

To close the above mentioned gap and provide a comprehensive approach to dropout intentions, the university dropout intention formation process instrument (UDIFP-29) was developed. The UDIFP-29 integrates theoretical elements from several models, including Tinto’s integration framework [32], Mashburn’s model of dropout awareness [36], and the Student Dropout Intentions Process Model proposed by Bäulke et al. [48].

Building upon this framework, the UDIFP-29 adopted a similar sequential logic but introduced key innovations. In the initial phase, it assessed specific sources of dissatisfaction across three critical domains: academic, social, and career-related. In the subsequent phase, it distinguished between two interconnected yet conceptually distinct psychological constructs: Ideation, defined as sporadic or recurring thoughts about leaving university, and Intention to dropout, understood as a more deliberate and planned decision to dropout studies.

Unlike Mashburn’s model [36], which considers ideation as a mediator between satisfaction and dropout behavior, the UDIFP-29 emphasizes a more detailed and staged structure of the process, in which each phase (dissatisfaction → ideation → intention) is evaluated as a distinct but related component within a dynamic process. This allows for a more specific and complex understanding of the development of quit intention, as well as greater precision in its measurement.

By synthesizing and extending previous models, the UDIFP-29 provides a novel contribution to the field: a culturally relevant, theoretically informed, and psychometrically validated instrument that captures the formation of dropout intention among first-year university students in Latin America.

In addition, to addressing the following significant gaps in the literature: the absence of a culturally validated, theoretically grounded, and psychometrically sound instrument that captures the formation of dropout intention during the critical early stages of university life, the UDIFP-29 conceptualizes dropout intention as a multidimensional, sequential process and adapts it to the realities of Chilean students.

### Research objectives of this study

**Fig 1:**
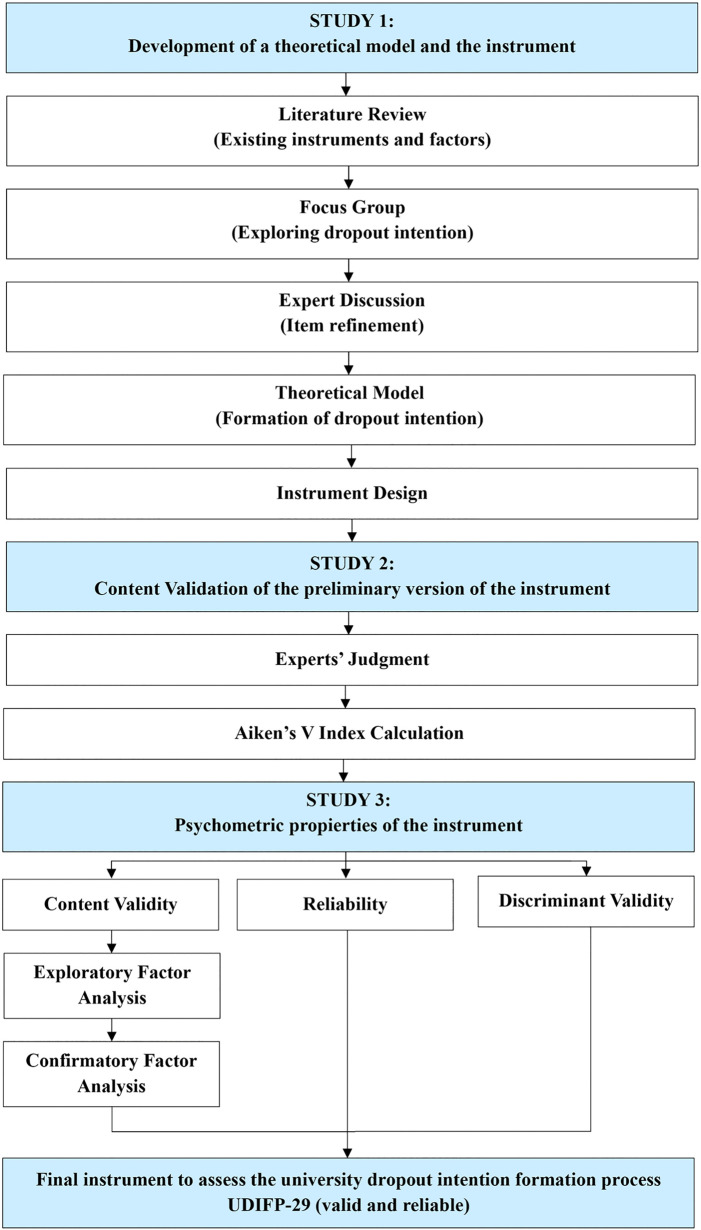
Process of Developing and Validating the Dropout Intention Instrument.

Intention to dropout plays an important role in the decision to quit university studies. In addition, identification of dropout intention is highly needed for the development of retention programs for students at universities. Therefore, it is pertinent to develop an instrument with evidence of validity and reliability that has the capacity to account for the process of formation of the intention to dropout. This is particularly important given the lack of psychometrically sound tools available. Therefore, the main objective of this research was to construct and validate a multidimensional self-report instrument to assess the process of forming the intention to dropout from the university. To achieve this objective, three studies were conducted (see Fig 1)., each with a different specific objective: (1) to construct a theoretical model that explains the dropout intention formation dropout intention phasing process in university students; (2) to provide content validity of the preliminary version of the instrument, and (3) to provide evidence of validity of the factor structure of the instrument.

### Method

The procedure for constructing and validating the instrument followed the guidelines of Carpenter [56], the International Test Commission [57], the American Psychological Association, the American Educational Research Association, and the National Council on Measurement in Education [58]. These guidelines provide recommendations for developing and validating scales. Three phases were conducted to design and validate the instrument: (1) Development of a Theoretical Model and Design of the instrument, (2) Content validation of the instrument by means of expert judgment, and (3) Estimation of psychometric properties. This research was approved by the Ethics, Bioethics and Biosafety Committee, coded as CEBB1394–2023. The instrument was constructed and validated through a two-year iterative process that included three studies. The following section describes these studies.

### Study 1: Development of a theoretical model and an instrument on the process of formation of university dropout intentions

The following strategies were employed to develop the theoretical model and the instrument: (1) conducting a systematic review of the literature; (2) holding focus groups and expert discussions; (3) constructing the theoretical model; and (4) designing the instrument. The procedure for each strategy is explained below.

First, a systematic review of the literature on dropout intentions was conducted to examine extant instruments and generate an initial set of items. This phase also highlighted the lack of tools addressing the formation of dropout intentions, thereby justifying the development of a new instrument. Secondly, focus groups were conducted to understand how the idea of dropping out formed among first-year students and which factors predominantly caused it. Subsequently, discussions were held with experts in the field (the authors of this paper and other colleagues researching dropout), who helped refine the items generated in the previous phase. Third, a theoretical model was formed using the information obtained from the literature review, the focus groups and the discussions with experts. This model served as the conceptual framework for the instrument, ensuring that each item aligned with the theoretical dimensions. Fourthly, the instrument was designed to include items corresponding to each dimension of the model. Both new and adapted items were included. Finally, the expert panel reviewed the final version through interviews and feedback rounds, allowing for adjustments to improve the clarity and contextual relevance of the instrument. Data collection started on March 4, 2023 and ended on July 30, 2024. The main results associated with these strategies are summarized below.

### Focus group

This study used a constructivist paradigm and a qualitative approach with a phenomenological design to understand how the intention of dropout arises out of the university experience, emphasizing the individuals and their subjective experiences.

### Participants

Three independent focus groups were formed. Participants were first-year psychology students from the same university in Chile, aged between 17 and 19 years. All participants were recruited through convenience sampling using announcements within the university department. The first group included five students, while the second and third groups had four participants each. Each group participated in three meetings throughout the semester: one at the beginning, one in the middle, and one at the end. In total, nine sessions were conducted (three per group) to ensure data saturation and comprehensive coverage of perspectives.

### Procedure for data collection and analysis

Focus groups were used to collect data on students’ perceptions and opinions regarding the formation of the intention to dropout at three key moments: the first academic semester (in the first weeks, before the exams), at mid-term (after the exams), and at the end of the semester. The focus group script was designed to explore the university experience. To prevent influencing students’ opinions, the script did not ask about intention to dropout, as it was expected that elements of the theoretical model would emerge spontaneously.

During the first session, the objectives of the research were clarified, written informed consent was obtained, and a duration of 45 minutes for each session was established. It was emphasized that there were no right or wrong answers, and that personal opinions were valued. Additionally, permission was requested to record the session. Participants were asked to speak one at a time and to raise their hand if they wished to express their opinion. To encourage interaction, icebreaker questions were included. Participants were asked to share the reasons why they were motivated to join the focus group and to explain why they chose their university program. These questions were intended to promote participation and help attendees feel more comfortable.

In the initial session, participants were asked to answer four questions. The first question asked about their overall experience during the first few weeks of classes. The second question asked about their feelings during the first week. The third and fourth questions asked about the main challenges they had faced so far and how they had managed to overcome them. In the second and third sessions, the same questions were revisited with a temporal focus: “Reflecting on these questions since our last session, how has your response changed?” The aim was to capture changes and developments in the students’ experiences and perceptions as the semester progressed. Content analysis was conducted manually, without the use of software.

### Results

In the first meeting, across all three groups, students reported feeling excited, fascinated, and anxious during the first weeks, as they adapted to the dynamics of university life. The students had mixed emotions about their new social relationships and felt apprehensive about the academic, social, and extracurricular challenges that they would have to manage. Managing new responsibilities and time effectively were prominent challenges. Entering a new academic stage after high school can evoke a complex set of emotions, often fueled by uncertainty about the future.

In the social area, participants emphasized the crucial importance of the welcome provided by second-year students during the first weeks of classes, indicating that it helped them feel integrated into their program and university life. As the days went by, there was a noticeable improvement in their relationships with their classmates, consolidating the formation of bonds of affection and a spirit of companionship among them. In addition, the appearance of social comparisons was highlighted, as students tended to contrast their performance with that of their peers, noting that some seemed to have a better understanding of the contents of the subjects. In summary, the social support received from peers and teachers was significantly valued. The disposition to companionship and mutual help, as well as the motivational support of teachers, contributed to improving the perception of social support and social connection in the initial stage of their academic trajectory. This is important as research has shown that both perceived social support and connection can positively influence motivation and counteract anxiety [59]. Anticipation of unfavorable test results (negative expectations) undermine motivation and alter study strategies, indicating that pre-existing predispositions and beliefs have a significant impact on academic performance. These expectations, when internalized, can create a self-perpetuating cycle of unsatisfactory results.

In the second meeting, after the first evaluations, across all three groups, it was observed that perceptions began to change. Students demonstrated the appearance of several emotions, such as happiness and gratitude for being in a career they like; alongside anxiety and stress in response to increased academic workload and performance evaluations.

Most students showed greater awareness and reflexivity in improving their self-regulation of learning; for example: study habits, study pace, use of efficient cognitive and metacognitive strategies for learning, the importance of balance between studies and personal life, prioritization of tasks, time planning, and so on. Exam pressure and expectations about results also began to influence study behavior, adaptation to university life and perception of academic stress. Research as shown that the number and difficulty of academic tend to increase over the course of the semester [60].

Lastly, in the third meeting at the end of the semester, some students expressed confidence and satisfaction in their chosen career, while two other demonstrated greater reflexivity and questioning about whether they had chosen the right career, whether they felt comfortable with their decision and whether they would continue with their studies due to the demands of their program.

In summary, at the first meetings, students mostly expressed thoughts and reflections linked to their desire to stay in their career. In contrast, during the second and third meetings, some students began to show intentions of dropout. Overall, a clear progression was observed in the first-year university experience. Initially, all students were engaged and motivated to complete their studies, but as time progressed, those students who reported greater social satisfaction with their career and performance wanted to remain at the university, while those who perceived low satisfaction presented dropout ideations that could lead to the intention of dropout.

### Theoretical model of process of formation of university dropout intention

The theoretical model of the university dropout intention formation process was designed to address a significant gap observed in the literature: the lack of a conceptual framework that described the evolutionary nature of dropout intention.

**Fig 2:**
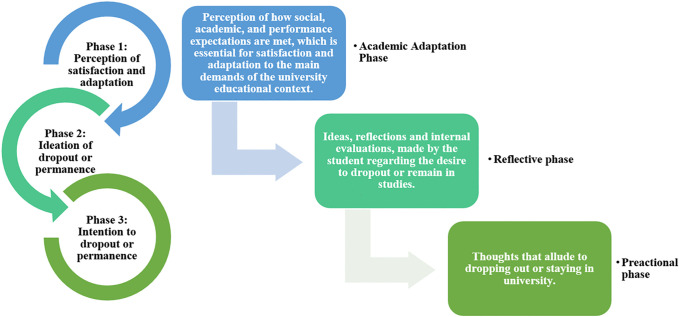
Theoretical model of process of formation of university dropout intention.

For its construction, we considered the results from a systematic literature review and focus groups with first-year students. Finally, we worked with a panel of experts to integrate all the information and shaped a model that reflected a sequential and dynamic process. The expert input was gathered from a panel of five researchers with extensive experience in higher education research and student retention. These experts were selected based on their publication record and involvement in related national research projects. They were consulted through semi-structured interviews and iterative feedback rounds on the initial model and item pool. Their insights contributed to refining the conceptual structure, ensuring theoretical coherence and contextual relevance to the Chilean higher education system (Fig 2).

The intention to dropout includes thoughts of leaving the university stemming from ideas, desires, reflections and internal evaluations that a student makes based on their perception of whether they have met social, academic, and performance expectations, which are considered essential for adapting to the main demands of the university context.

The process of formation of dropout intention is structured through three interconnected phases that function dynamically: (Phase 1) Perception of satisfaction and adaptation; (Phase 2) Ideation of dropout; and (Phase 3) Intention to dropout. Each phase metacognitively represents the processes occurring in the students’ minds that are reflected in their behavior.

Phase 1 is adaptive, associated with satisfaction with new experiences and the constant adjustment necessary for effective academic and social adaptation in their chosen career. Phase 2 involves reflection on lived experiences. The term “reflective” refers to a process of deep analysis, in which various aspects of a situation are carefully considered. Phase 3 is pre-actional, characterized by the planning and anticipation of actions. In this context, a reflective phase can lead to a pre-actional phase, in which intentions are formed and subsequent actions are prepared.

### Design of the instrument

The instrument’s design was based on the proposed theoretical model, which offers a comprehensive view of the dropout intention formation process. It integrates two dimensions that are typically analyzed separately. The first perspective, incorporates three key factors that traditionally influence university adjustment and the decision to dropout: Social Satisfaction (a student’s perception of fulfilled social expectations), Career Satisfaction (the perceived fulfillment of expectations related to the academic program and professors), and Academic Performance Satisfaction (the perceived fulfillment of performance expectations linked to learning behaviors and effective learning strategies). Second perspective, closely related to the above, examines the internal process of a student’s desire to dropout. It distinguishes between Ideation (initial thoughts and reflections about leaving studies) and Intention to dropout (concrete thoughts alluding to dropping out of higher education). From a process-oriented perspective, these two perspectives allow for an understanding of the underlying sequence in the formation of dropout intention, which develops through three distinct phases: dissatisfaction, ideation, and intention.

For its construction, a database was created in Microsoft Excel 2021, which allowed for a systematic process of item selection, adaptation, and creation. The items were derived from the results of the systematic literature review and aligned with the study’s theoretical framework. Generation and refinement of the items were conducted through discussion and reflection sessions exclusively among the lead researchers, guided by theoretical coherence, relevance to the construct of dropout intention, and consensus on the clarity and scope of each item. Finally, the items were organized according to the model’s dimensions, reflecting perceptions of satisfaction and adaptation (social satisfaction, career satisfaction, and academic performance satisfaction) as well as the process phases (ideation and dropout intention).

The purpose of these sessions was to ensure the instrument’s theoretical coherence and the relevance of each item to the dropout intention construct. Consensus among the researchers guaranteed that the meaning and scope of each item were clear and consistent with the proposed model.

Four previously validated items that align with the theoretical model were incorporated. These were selected from open-access instruments, ensuring their suitability for use in the present research: (1) “I am satisfied with the quality of the subjects I have”, corresponding to item A43 of the Student Adaptation to University Questionnaire (SACQ) [61]; (2) “I am satisfied with the teachers I have”, corresponding to item A62 of the Student Adaptation to University Questionnaire (SACQ) [61]; (3) “I would recommend this career to a friend”, which is item 6 of the “Screening instrument for students at-risk of dropping out from Higher Education” [51]; and (4) “I am thinking of leaving university for good”, item I60 of the Student Adaptation to University Questionnaire (SACQ) [61]. The remaining items were adapted or designed specifically for this research, ensuring comprehensive coverage of the dimensions of the model.

This process resulted in the conformation of an initial version of the instrument, composed of 31 carefully selected items and organized in coherence with the theoretical model, as illustrated in Table 1. Finally, this preliminary version was subjected to a content validation process, which included an evaluation by experts and a cognitive interview to obtain a first impression of its reliability and applicability.

**Table 1:** Initial Version: Dropout Intention Formation Process Instrument.

Phases	Conceptualizations		Items
**Phase 1: Perception of satisfaction and academic adaptation: Perception of the fulfillment of social, academic and performance expectations essential for satisfaction and adaptation to the main demands of the university context.**	1.1. Social satisfaction: Perception of the fulfillment of expectations related to social relations in the academic context.	1	I have close personal relationships with my career classmates.
		2	I am adapted to the social environment of the career.
		3	I feel that I integrate with my classmates.
		4	I have good relationships with my classmates.
		5	I am satisfied with my social adjustment in my career.
	1.2. Career satisfaction: Perception of the fulfillment of expectations regarding the subjects, contents and professors.	6	I am satisfied with the number of subjects I have.
		7	I am satisfied with the quality of the subjects I have.
		8	I am satisfied with the teachers I have.
		9	I am satisfied with the contents of the subjects I have.
		10	I am very interested in the content covered in the classes.
		11	I would recommend this career to a friend.
	1.3. Satisfaction with academic performance: Perception on the fulfillment of performance expectations in the career, linked to academic behaviors and active strategies to ensure effective learning.	12	I am satisfied with the outcome of the evaluations.
		13	I am satisfied with the learning I am achieving.
		14	I am satisfied with the way I am studying.
		15	I am satisfied with the academic goals I set for myself (e.g., getting a high grade).
		16	I am satisfied with my academic to-do list.
		17	I am satisfied with the organization of the academic tasks I perform.
		18	I am satisfied with the learning goals I set for myself.
		19	I am satisfied with the time I spend studying.
**Phase 2: Ideation of dropout: Ideas, reflections and internal evaluations made by the student regarding the desire to drop out studies.**	Passive Dropout Ideation: Unsystematic, scattered and fanciful ideas regarding the desire to drop out university.	20	I have been wishing for something to happen that would free me from going to university and/or studying.
		21	I have felt like not getting up to avoid going to university.
		22	In short, I would rather not be studying at the university.
	Active Ideation of Dropout: Systematic and structured ideas involving desires to drop out. It involves weighing the advantages and disadvantages, as well as the impact of dropping out university.	23	I have thought about what I could do instead of studying for getting my degree.
		24	I have been undecided about whether to continue studying this career.
		25	I can’t get rid of the feeling that I should give up my career.
		26	I am evaluating what it would mean for me to dropout.
		27	I am evaluating the advantages and disadvantages of dropping out.
		28	I am reflecting on the short- and long-term consequences of dropping out.
**Phase 3: Intention to dropout: Thoughts that allude to dropping out of higher education.**	Dropout Intention: Thoughts that allude to dropping out university.	29	I am thinking of dropping out higher education.
		30	I am thinking of dropping out university for good.
		31	I have thought about the possibility of dropping out of higher education.

## Study 2: Content validity of the preliminary version of the instrument on the process of formation of university dropout intentions

### Method

The methodology of expert judgment was used for the content validation process.

### Participants

Five expert judges participated in the content validation process, four Chilean and one Portuguese, two women and three men. All met the following inclusion criteria: (1) doctoral degree in psychology, education, or related areas; (2) experience and knowledge in the areas of social science research methodology and/or educational psychology; (3) experience in the construction of measurement instruments.

### Procedure for data collection and analysis

Specialists were invited by e-mail to participate in the study as judges. The invitation included details about the study’s context and description, its objective, and the role they would play in this process. In addition, indicators and standards for the evaluation, as well as detailed descriptions of each factor to be assessed within the questionnaire, were included. The expert judges were asked to assess and score each item of the instrument according to four essentials levels: a) sufficiency (evaluation of whether the items were adequate for the measurement), b) clarity (syntactic and semantic understanding), c) coherence (logical consistency with the factor evaluated), and d) relevance (importance and need for inclusion in the study). Three levels were established: (1) low level; (2) medium level; and (3) high level. Judges were allowed to make additional comments next to each item.

Qualitative and quantitative analyses were performed based on the judges’ scoring performance. In situations where the judges did not agree on the evaluation of an item, the item was modified. Content validity was assessed using Aiken’s V index, which measures the degree of agreement among expert judges. A minimum value of 0.8 was established as the criterion for acceptance [62]. Additionally, asymmetric 95% confidence intervals were calculated for Aiken’s V, considering values greater than 0.5 at the lower limit as acceptable [63].

### Results

The results of the content validity of the preliminary version of the instrument are shown in Table 2.

**Table 2:** Aiken’s V Index Values for Each Scale Item.

			Clarity		Consistency		Relevance		Sufficiency	
		Item	Aiken’s V	95% CI	Aiken’s V	95% CI	Aiken’s V	95% CI	Aiken’s V	95% CI
**Phase 1: Perception** **of Satisfaction** **and Adaptation**	***Social Satisfaction***	1	0.9	[0.69 - 1]	1	[0.84- 1]	1	[0.84- 1]	1	[0.84- 1]
		2	0.8	[0.57 - 1]	0.9	[0.69 - 1]	1	[0.84- 1]	1	[0.84- 1]
		3	0.9	[0.69 - 1]	1	[0.84- 1]	1	[0.84- 1]	1	[0.84- 1]
		4	1	[0.84- 1]	1	[0.84- 1]	0.9	[0.69 - 1]	1	[0.84- 1]
		5	0.8	[0.57 - 1]	1	[0.84- 1]	1	[0.84- 1]	1	[0.84- 1]
	***Career Satisfaction***	6	1	[0.84- 1]	1	[0.84- 1]	0.9	[0.69 - 1]	1	[0.84- 1]
		7	0.9	[0.69 - 1]	1	[0.84- 1]	1	[0.84 - 1]	1	[0.84- 1]
		8	0.8	[0.57 - 1]	1	[0.84 - 1]	1	[0.84 - 1]	1	[0.84- 1]
		9	0.8	[0.57 - 1]	0.9	[0.69 - 1]	1	[0.84 - 1]	1	[0.84- 1]
		10	1	[0.84 - 1]	1	[0.84 - 1]	1	[0.84 - 1]	1	[0.84- 1]
		11	1	[0.84 - 1]	1	[0.84 - 1]	1	[0.84 - 1]	1	[0.84- 1]
	***Performance Satisfaction***	12	1	[0.84 - 1]	1	[0.84 - 1]	1	[0.84 - 1]	1	[0.84- 1]
		13	1	[0.84 - 1]	1	[0.84 - 1]	1	[0.84 - 1]	1	[0.84- 1]
		14	0.8	[0.57 - 1]	0.6	[0.36 -0.97]	0.8	[0.57 - 1]	1	[0.84- 1]
		15	0.7	[0.46 - 1]	0.8	[0.57- 1]	0.9	[0.69 - 1]	1	[0.84- 1]
		16	1	[0.84 - 1]	0.8	[0.57- 1]	0.9	[0.69 - 1]	1	[0.84- 1]
		17	0.8	[0.57 - 1]	0.6	[0.36 – 0.91]	0.8	[0.57 - 1]	1	[0.84- 1]
		18	1	[0.84 - 1]	1	[0.84 - 1]	0.8	[0.57 - 1]	1	[0.84- 1]
		19	1	[0.84 - 1]	1	[0.84 - 1]	0.8	[0.57 - 1]	1	[0.84- 1]
**Phase 2: Ideation** **of dropout** **or permanence**	***Passive dropout ideation***	20	0.9	[0.69 - 1]	0.9	[0.69 - 1]	0.9	[0.69 - 1]	1	[0.84- 1]
		21	1	[0.84 - 1]	0.9	[0.69 - 1]	0.9	[0.69 - 1]	1	[0.84- 1]
		22	1	[0.84 - 1]	1	[0.84 - 1]	1	[0.84 - 1]	1	[0.84- 1]
	***Active*** ***dropout ideation***	23	1	[0.84 - 1]	1	[0.84 - 1]	1	[0.84 - 1]	1	[0.84- 1]
		24	1	[0.84 - 1]	1	[0.84 - 1]	0.8	[0.57 - 1]	1	[0.84- 1]
		25	1	[0.84 - 1]	1	[0.84 - 1]	1	[0.84 - 1]	1	[0.84- 1]
		26	0.9	[0.69 - 1]	1	[0.84 - 1]	0.9	[0.69 - 1]	1	[0.84- 1]
		27	1	[0.84 - 1]	1	[0.84 - 1]	1	[0.84 - 1]	1	[0.84- 1]
		28	0.9	[0.69 - 1]	1	[0.84 - 1]	0.7	[0.46 - 1]	1	[0.84- 1]
**Phase 3: Intention to dropout** **or permanence**	***Dropout Intention***	29	1	[0.84- 1]	1	[0.84 - 1]	1	[0.84 - 1]	1	[0.84- 1]
		30	1	[0.84 - 1]	1	[0.84 - 1]	1	[0.84- 1]	1	[0.84- 1]
		31	0.9	[0.69 - 1]	1	[0.84 - 1]	1	[0.84- 1]	1	[0.84- 1]

Regarding sufficiency, the items reached maximum values in the Aiken’s V index and in the confidence intervals. This indicates that the dimensions possess adequate coverage, ensuring that the items are collectively sufficient to measure the proposed factors.

Regarding clarity, the validation process followed an interactive approach. In phases 1, 2 and 3 all the items obtained values higher than 0.8; and in the lower limits of their confidence intervals, they obtained indices higher than 0.5; except item 4 of the academic performance factor, which was revised and modified. Consistently, in phase 1, most of the items obtained values higher than 0.8; and in the lower limits of their confidence intervals, they obtained indices higher than 0.5; except for items 3 and 6 of the academic performance factors, which were revised and modified. In phase 2, all items obtained values higher than 0.9; and in the lower limits of their confidence intervals, they obtained indices higher than 0.5. Finally, in phase 3 all items demonstrated optimal indices, confirming the instrument’s clarity and formal stability.

Regarding relevance, the items consistently achieved Aiken’s V values above 0.8 across all three phases and in the lower limits of their confidence intervals, they obtained indexes higher than 0.5; except for item 6 of the active dropout intention factor, which was revised and modified. Following these adjustments, all items in phase 3 yielded optimal indices. The materials used in the expert assessment are available and can be sent upon request to the authors of this research.

The findings of the content validation by expert judgment for the initial version of the instrument highlight several characteristics and offer a generally favorable assessment. However, as indicated, some items scored slightly lower than desired Aiken’s V, with rates which did not reach the minimum expected at the lower limits of their confidence intervals. This led to the modification and restructuring of these items, for which the qualitative comments made by the judges in the document were also considered. After these modifications, the items were not re-evaluated by the expert judges.

### Validity based on response processes: Cognitive interviews

After making the necessary modifications to the instrument according to the recommendations of the experts, cognitive interviews were conducted as another source of validation. These interviews followed a *think aloud* protocol, in which students were asked to verbalize their thoughts as they responded to the instrument’s items. Students were asked for informed consent and authorization to record their responses. These were transcribed and analyzed. The purpose of this methodology was to identify and correct difficulties related to the instructions, the clarity of the questions, the items, and the format of the instrument’s answers. All this was done to evaluate the items’ comprehensibility and to assess the viability of the instrument from the students’ interpretation perspective [64].

### Participants

The participants were five first-year students in their first semester recruited through convenience sampling. They belonged to the programs of Obstetrics, Commercial Engineering, Industrial Civil Engineering and Psychology at an urban, public university in southern Chile, as well as Kinesiology students at a private university. All participants were between 18 and 19 years old and signed written informed consent forms prior to participating. The interviews lasted between 10 and 15 minutes, with the longest interview lasting 29 minutes for a Psychology student, which may reflect greater reflection or discussion on psychological topics or simply individual variations during the interviews.

### Results

Analysis of the data from the cognitive interviews revealed several important aspects of item comprehension and wording. For most of the items, no modifications were necessary since the subjects reported them to be easy to read and understand. However, comprehension difficulties were observed with the word *socially* in item 2; which was revised to: *I am adapted to the social environment of the career.* In item 17, the word *planning* was replaced with *organization*, resulting in the revised item: *I am satisfied with the organization of the academic tasks I perform.* Overall, most items were well understood and did not require modifications.

## Study 3: Psychometric Properties of the Instrument on the process of formation of university dropout intentions

### Method

A psychometric study was carried out with the aim of estimating validity and reliability indices of the instrument.

### Participants

A non-probability convenience sampling procedure was used for participant recruitment. The sample consisted of 678 first-year students from the same Chilean university. The average age was 18.86 years (SD = 1). Of these, 379 were men, 272 were women, 7 identified themselves with other genders, and 20 students preferred not to answer. As for the distribution by academic program, 24 students came from Economics and Administrative Sciences, 306 from Medicine, 299 from Engineering, 14 from Biological Sciences, 23 from Physical and Mathematical Sciences, and 12 from Chemistry.

### Procedure for data collection and analysis

Data collection took place in person, in classroom settings, with students physically present in the same room. At the beginning of each session, participants were verbally informed about the study’s objectives and the ethical safeguards in place, including the voluntary nature of participation and the possibility of asking questions. Participation was anonymous and confidential. Afterwards, printed informed consent forms were distributed and signed by the participants, followed by the paper-based administration of the questionnaires.

A descriptive statistical analysis of the items was carried out to evaluate their normal distribution, skewness, and kurtosis coefficients. Factor analyses were performed to determine construct validity by dividing the sample into two parts: one part (n = 463) for the exploratory factorial analysis (EFA) and the other part (n = 215) for the factorial confirmatory analysis (CFA).

The EFA was used to obtain preliminary evidence about the factorial structure of the instrument (31 items) while the CFA was carried out to corroborate this structure. Parameters were determined using the robust maximum likelihood estimator (MLR) for continuous variables. This estimation method is effective even without the need to comply with the assumptions of normality, since its robustness allows the identification of existing effects in a reliable manner [65]. An oblique Geomin rotation was used, as correlations among latent factors were expected. The number of factors was initially guided by the theoretical model and then confirmed by goodness-of-fit indices. The factor structure underlying a correlation matrix for each latent variable was evaluated through the CFA. The adjustment indices of the model proposed in the literature [65] served to determine the best model; these indices are: (1) Chi-square values (*X*^2^) not significant p ≥ .05, (2) root mean squared error of approximation (RMSEA) values less than.07, (3) comparative adjustment index (CFI) and unstandardized adjustment index (TLI) should be greater than.94, and (4) the factor loadings of the items must be significantly equal to or greater than.40. In addition to the standard fit indices, Akaike (AIC) and Bayesian (BIC) information criteria. The MPlus 8.4 software was used.

Reliability was calculated using alpha and McDonald (1978) Omega coefficient (ω); this method is more accurate for determining reliability because, unlike alpha, its estimation is based on factor loadings and not on the number of items or response options [66]. The free software JASP v. 0.8.3.1 was used. Composite reliability (CR) was also calculated to assess the internal consistency of the latent constructs, given that this index does not assume tau equivalence, unlike alpha, and is more appropriate for confirmatory factor models. CR values were estimated from the standardized factor loadings and their respective error variances, according to the recommendations of literature [65]. Values above.70 were considered adequate, and those above.90 were considered excellent.

To confirm construct validity, both convergent validity and divergent validity were tested. To do this, AVE (Average Variance Extracted) comparison criteria [67] were used for each first-order factor of the instrument. AVE values are expected to be greater than.50.

To assess for divergent validity, the mean scores of each subscale were correlated with the “Willpower” subscale from the *Brief Scale Motivation Regulation* developed by Kim et al. [68]. It evaluates the general tendency of university students to self-regulate their motivation and comprises two subscales: Motivation Regulation (8 items), which assesses students’ strategic efforts to sustain motivation, and Willpower (4 items), which evaluates persistence strategies used when facing uninteresting or difficult tasks. In this investigation, only the “Willpower” factor was used. This self-report instrument rated on a Likert-type scale ranging from 1 (never) to 7 (very frequently). Divergent validity implies that two theoretically unrelated variables are also empirically unrelated, reflected in low or non-existent correlations. In this research, which explores validity based on divergent measures, correlations of magnitude less than.30 are expected, as an indicator that the instruments measure distinct constructs.

Additionally, we considered evaluating the square root of the AVE and comparing it with the correlation of each subscale, using a scale that measures a different construct in theoretical terms: Willpower [68] hoping to find correlations lower than the square root of the AVE in order to meet this validity criterion [67]. The free software JASP v. 0.8.3.1 was used.

### Results

The analysis of skewness and kurtosis evidence that all items were within the theoretically adequate range of −2 and +2. These results suggest that the data distribution approximates normality for the majority of the scale. However, items 29 and 30 exceeded these thresholds, exhibiting a leptokurtic distribution characterized by a more pronounced peak and thicker tails compared to a normal distribution. The analysis is represented in Table 3.

**Table 3:** Descriptive Statistics of the Instrument’s Items.

	Mean	Deviation	Skewness		Kurtosis	
	Value	Value	Value	Standard Error	Value	Standard Error
1	4.59	2.133	−0.488	0.094	−1.160	0.188
2	5.09	1.570	−0.743	0.094	−0.167	0.187
3	5.00	1.741	−0.752	0.094	−0.388	0.188
4	5.38	1.540	−1.048	0.094	0.651	0.187
5	4.90	1.715	−0.734	0.094	−0.249	0.188
6	5.05	1.512	−0.671	0.094	0.056	0.188
7	4.96	1.493	−0.717	0.094	0.173	0.188
8	4.92	1.549	−0.716	0.094	−0.031	0.188
9	4.80	1.504	−0.700	.094	0.158	0.188
10	5.32	1.344	−1.083	0.094	1.279	0.187
11	5.10	1.668	−0.868	0.094	0.147	0.188
12	3.57	1.784	0.049	0.095	−1.055	0.189
13	4.38	1.684	−.510	0.094	−0.595	0.188
14	3.78	1.689	−0.040	0.094	−0.898	0.187
15	4.43	1.731	−0.388	0.094	−0.759	0.188
16	3.96	1.580	−0.182	0.094	−0.563	0.188
17	3.98	1.635	−0.135	0.094	−0.750	0.187
18	4.29	1.651	−0.405	0.094	−0.561	0.188
19	3.69	1.712	−0.053	0.094	−0.940	0.188
20	3.15	2.134	0.526	0.094	−1.155	0.188
21	4.39	2.167	−0.356	0.094	−1.263	0.188
22	2.25	1.651	1.314	0.094	0.812	0.187
23	3.41	2.152	0.270	0.094	−1.377	0.188
24	2.87	2.061	0.699	0.094	−0.927	0.188
25	2.74	2.136	0.829	0.094	−0.835	0.188
26	2.50	1.978	1.051	0.094	−0.293	0.188
27	2.75	2.107	0.810	0.094	−0.849	0.187
28	2.10	1.721	1.518	0.094	1.159	0.188
29	1.65	1.333	2.371	0.094	5.178	0.187
30	1.50	1.162	2.756	0.094	7.758	0.188
31	1.99	1.695	1.738	0.094	1.878	0.188

### Exploratory factor analysis

To assess the factor structure of the instrument, an EFA was performed using Mplus with the Robust Maximum Likelihood (MLR) estimator and Geomin oblique rotation, as correlations between factors were expected.

The Kaiser-Meyer-Olkin (KMO) test yielded a value of 0.771, indicating acceptable suitability for factor analysis. Additionally, Bartlett’s test of sphericity was significant (χ²(15) = 1276.81, p < .001), confirming the appropriateness of performing the factor analysis.

**Fig 3:**
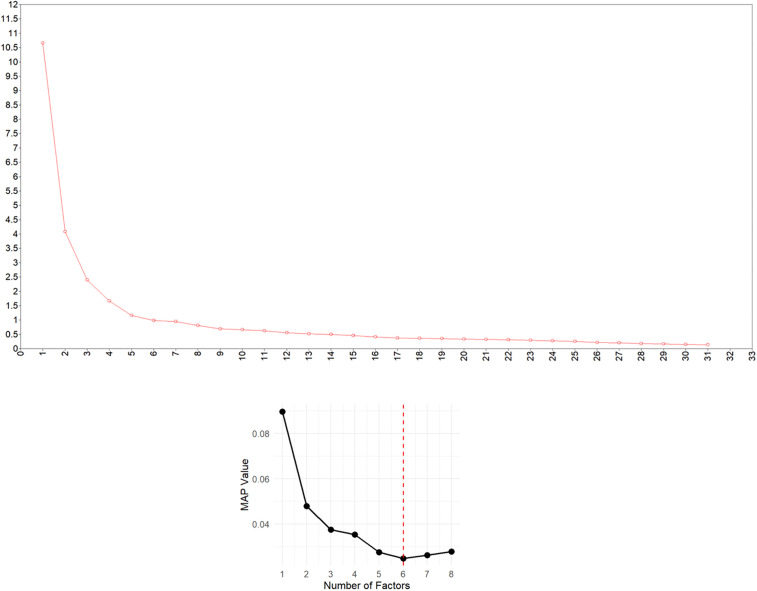
Scree plot and MAP test for determining the number of factors and items to retain.

The number of factors to retain was determined using both empirical and theoretical criteria. First, we examined the eigenvalues and applied Kaiser’s criterion (eigenvalues greater than 1), and then inspected the scree plot to identify the inflection point. The scree plot (Fig 3) showed a clear “elbow” at the sixth factor, suggesting that a six-factor solution was optimal and consistent with the theoretical structure of the instrument.

To complement these criteria, we also computed Velicer’s Minimum Average Partial (MAP) index. The MAP values decreased steadily from one to six factors and reached their minimum at the six-factor solution, after which they increased again for seven and eight factors. According to Velicer’s criterion, this pattern indicates that the six-factor solution provides the most parsimonious representation of the data. Thus, converging evidence from Kaiser’s criterion, the scree plot, the MAP index (also displayed in Fig 3a), and the theoretical model all supported the retention of six factors.

Two models were estimated, a 5-factor model and a 6-factor model. The fit indices for both models are presented in Table 4. The fit indices for the 6-factor model were: χ²(294) = 551.03, p < .05; CFI = .962, TLI = .940, RMSEA = .043 (90% CI [.038 −.049]), and SRMR = .021. These values indicate a good fit of the model to the data, following the guidelines of Hu and Bentler [69], which suggest that CFI and TLI > .95, RMSEA < .06, and SRMR < .08 are indicators of a good fit.

**Table 4:** Estimates from the Exploratory Factor Analysis Model.

	X2	Df	RMSEA	(90% CI)	SRMR	CFI	TLI
**5-factor model**	650.492*	320	.047	.042−.052	.026	.951	.929
**6-factor model**	551.026*	294	.043	.038-049	.021	.962	.940

Df = degrees of freedom of the model; RMSEA = Root Mean Square Error of Approximation; (90% CI) = 90% confidence interval for RMSEA; SRMR = Standardized Root Mean Square Error of Approximation; CFI = Comparative Fit Index; TLI = Tucker-Lewis Index; *p < .05

To identify the items of each factor, significant items with loadings greater than.40 were retained. Items 10 and 11 were eliminated due to low loadings, resulting in a final model consisting of 29 items distributed across 6 factors. The details of the factor loadings are shown in Table 5.

**Table 5:** Loadings of the Items in the Factors of the Instrument.

Item	Factor 1	Factor 2	Factor 3	Factor 4	Factor 5	Factor 6
1	0.447*	0.070	0.000	0.021	−0.007	0.029
2	0.737*	−0.007	0.026	−0.178	−0.031	0.047
3	0.922*	−0.021	0.025	0.012	−0.018	0.007
4	0.829*	0.092	−0.044	0.054	0.051	−0.060
5	0.745*	−0.025	0.090	−0.057	−0.029	−0.037
6	0.079	0.685*	−0.002	−0.113	0.011	0.057
7	0.093*	0.679*	0.082	−0.067	0.027	−0.056
8	0.010	0.640*	−0.089	0.062	−0.056	−0.015
9	−0.063	0.791*	0.065	0.008	−0.067	−0.016
10	−0.054	0.321*	0.068	−0.153	−0.193	−0.039
11	0.072	0.152	0.144	−0.213*	0.005	−0.029
12	−0.003	0.082	0.699*	0.146	−0.125	0.042
13	0.001	0.299*	0.497*	0.041	0.043	0.009
14	−0.022	−0.082	0.912*	0.038	−0.027	−0.098*
15	0.099*	−0.002	0.748*	0.092	−0.058	−0.047
16	0.013	0.124*	0.724*	−0.118	0.064	0.070
17	0.004	0.031	0.793*	−0.083	0.146	−0.090
18	0.048	0.040	0.782*	−0.041	−0.015	0.002
19	0.007	−0.047	0.767*	−0.085	−0.035	0.044
20	−0.018	0.003	−0.071	0.527*	0.223	0.066
21	0.040	−0.027	−0.174*	0.426*	0.125	−0.016
22	−0.113*	−0.069	0.117	0.508*	0.029	0.272*
23	−0.010	−0.025	0.032	0.239*	0.606*	−0.023
24	0.042	−0.031	−0.072	0.189	0.655*	0.061
25	−0.036	0.042	0.027	0.012	0.881*	−0.007
26	0.013	−0.085	0.024	−0.047	0.814*	0.031
27	−0.053	0.071	−0.019	0.056	0.829*	0.020
28	−0.027	−0.051	−0.026	−0.013	0.605*	0.310*
29	−0.019	0.021	−0.045	0.045	−0.017	0.918*
30	−0.021	−0.065	0.019	−0.048	0.028	0.854*
31	0.046	0.032	−0.005	0.218	0.162	0.628*

*p < .05

Factor 1 grouped five items assessing the fulfillment of expectations related to social relationships in an academic context, which refers to Social Satisfaction. Factor 2, meanwhile, grouped four items measuring the degree of satisfaction with the content, subjects, and professors, reflecting Career Satisfaction. Factor 3 consisted of eight items addressing the fulfillment of expectations regarding academic performance, which is related to academic behaviors and active strategies to ensure effective learning, corresponding to Performance Satisfaction. Factor 4 includes three items measuring scattered, unsystematic, and fanciful thoughts about the desire to drop out of university, which is associated with Passive Dropout Ideation. Factor 5 consists of six items that assess more structured and deliberate thoughts about dropping out, considering the pros and cons, as well as the impact of leaving university, which corresponds to Active Dropout Ideation. Finally, Factor 6 consists of three items that focus on Dropout Intention, specifically referring to the intention to leave higher education.

### Confirmatory factor analysis

A CFA was performed to compare two alternative models and determine which presented a better fit to the data. The first alternative corresponded to a six-factor correlated structure (Solution 1): χ²(362) = 514.852*, p < .05; RMSEA = .044 [.035 –.053]; SRMR = 0.052; CFI = .949; TLI = .942; AIC = 21431.85; BIC = 21775.65. The second alternative consisted of a more parsimonious structure, in which the satisfaction factors (career satisfaction, performance satisfaction, and social satisfaction) were cluster on the one hand, and the ideation factors (passive and active ideation) on the other (Solution 2): χ²(369) = 524.936*, p < .05; RMSEA = .044 [.035 –.053]; SRMR = .057; CFI = .948; TLI = .942; AIC = 21429.24; BIC = 21749.45. See Table 6.

**Table 6:** Loadings standardized factor loadings of the items in the factors of the instrument.

Item	Factor 1	Factor 2	Factor 3	Factor 4	Factor 5	Factor 6
1	0.506 (0.059)					
2	0.770 (0.040)					
3	0.850 (0.041)					
4	0.795 (0.038)					
5	0.795 (0.042)					
6		0.674 (0.051)				
7		0.846 (0.037)				
8		0.537 (0.056)				
9		0.790 (0.037)				
10			0.755 (0.033)			
11			0.782 (0.039)			
12			0.818 (0.031)			
13			0.743 (0.040)			
14			0.809 (0.041)			
15			0.706 (0.047)			
16			0.794 (0.033)			
17			0.771 (0.040)			
18				0.698 (0.056)		
19				0.456 (0.080)		
20				0.636 (0.062)		
21					0.624 (0.051)	
22					0.845 (0.033)	
23					0.825 (0.029)	
24					0.882 (0.033)	
25					0.866 (0.026)	
26					0.873 (0.027)	
27						0.881 (0.036)
28						0.865 (0.036)
29						0.728 (0.069)

Standard error is included in parentheses.

**Fig 4:**
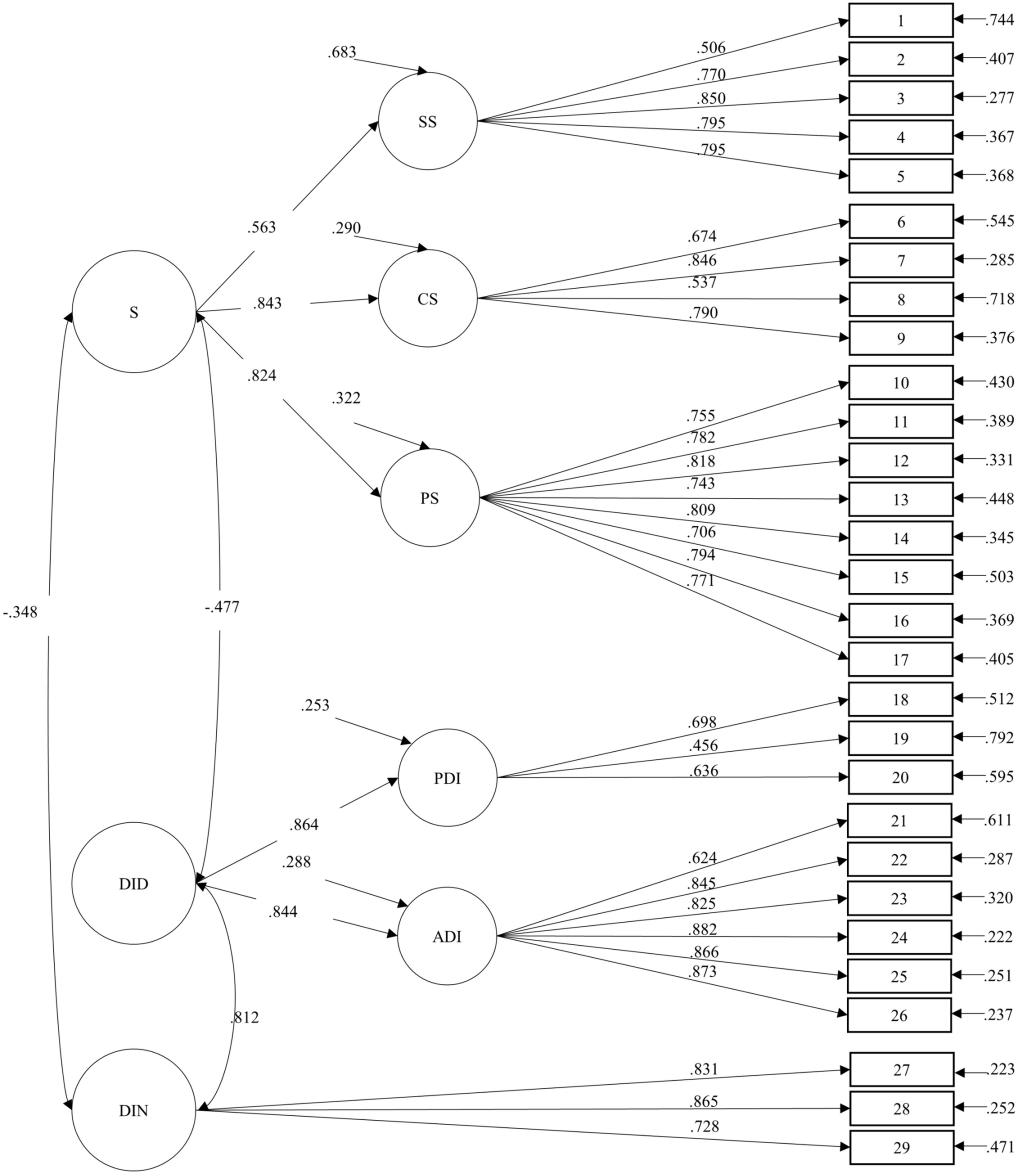
Indices of the Factorial Structure.

Hence, a model composed of 2 first-order and 6 second-order models was confirmed, see Fig 4. The first factor composed of 5 items refers to social satisfaction (SS). The second factor is composed of 4 items related to career satisfaction (CS) aspects. The third factor composed of 8 items groups items related to academic performance satisfaction (PS). These three factors constitute a general factor which, from a theoretical perspective, reflect the perception of satisfaction and adaptation regarding career, social and academic performance aspects (S). The fourth factor, composed of 3 items, refers to passive dropout ideation (PDI). The fifth factor, composed of 6 items, refers to active dropout ideation (ADI). These three factors constitute another general factor, which from a theoretical perspective, reflect the dropout ideation (DID). The sixth factor, composed of 3 items, refers to dropout intention (DIN). It is concluded that the structure obtained was confirmed in relation to the proposed theoretical model. The final instrument, a self-administered questionnaire, consisted of a total of 29 items. Participants’ responses were captured using a 7-point Likert scale to measure the specified constructs (see S1 Appendix).

### Reliability

The internal consistency analyses for each factor of the dropout intention instrument indicate adequate to excellent reliability, with most omega (ω) coefficients exceeding the recommended threshold of.80. Specifically, high internal consistency was observed for: Social Satisfaction (ω = .915), Career Satisfaction (ω = .826), Performance Satisfaction (ω = .925), and the Total of the three satisfaction factors (ω = .859). Regarding the dropout-related variables, Active Dropout Ideation (ω = .925), Total Dropout Ideation (ω = .908), and Dropout Intention (ω = .884) also demonstrated strong internal consistency. However, Passive Dropout Ideation showed a lower reliability index (ω = .680), suggesting caution in interpreting this scale independently. Overall, the results support the internal consistency and reliability of the scales in measuring the proposed dimensions related to dropout intention (see Table 7).

**Table 7:** Scales Reliability Indices and Central Tendency Measures.

Scale	Coef. ω	Error ω	IC ω (95%)	Coef. α	Error α	IC α (95%)	Mean	Error mean	CI Mean (95%)	SD	Error SD	CI SD (95%)
**Social Satisfaction**	0.915	0.006	0.904–0.925	0.917	0.005	0.906–0.926	4.529	0.043	4.445–4.613	1.090	0.027	1.034–1.153
**Career Satisfaction**	0.826	0.013	0.798–0.849	0.821	0.014	0.792–0.845	4.934	0.047	4.841–5.026	1.223	0.032	1.161–1.292
**Performance Satisfaction**	0.925	0.005	0.914–0.934	0.924	0.005	0.913–0.934	4.011	0.053	3.907–4.115	1.363	0.032	1.293–1.441
**Total of the three factors**	0.859	0.011	0.838–0.879	0.855	0.011	0.832–0.875	4.998	0.054	4.892–5.103	1.396	0.037	1.325–1.475
**Passive dropout ideation**	0.680	0.022	0.636–0.722	0.664	0.022	0.620–0.705	3.265	0.060	3.148–3.381	1.546	0.034	1.468–1.633
**Active dropout ideation**	0.925	0.005	0.914–0.935	0.924	0.005	0.912–0.934	2.719	0.067	2.589–2.850	1.728	0.042	1.640–1.826
**Total of both factors**	0.908	0.006	0.896–0.918	0.904	0.006	0.893–0.914	2.897	0.059	2.782–3.013	1.520	0.035	1.442–1.606
**Dropout Intention**	0.884	0.016	0.848–0.910	0.875	0.016	0.837–0.904	1.715	0.049	1.620–1.811	1.263	0.060	1.198–1.334

In addition to Cronbach’s alpha, composite reliability (CR) was calculated to assess the internal consistency of the latent constructs, addressing the limitations of alpha under the assumption of tau-equivalence. CR values were computed using standardized factor loadings and their corresponding error variances. In line with recommended thresholds, values above.70 were considered acceptable, and those exceeding.90 indicated excellent reliability. The results showed that Social Satisfaction (CR = 0.864), Career Satisfaction (CR = 0.809), Performance Satisfaction (CR = 0.923), Active Dropout Ideation (CR = 0.926), and Dropout Intention (CR = 0.866) demonstrated good to excellent internal consistency. However, the Passive Dropout Ideation factor presented a lower CR value (CR = 0.627), indicating potential issues in item coherence that may warrant future refinement. Overall, the composite reliability indices support the adequacy of the measurement model and reinforce the reliability of the latent constructs used in the analysis.

### Validity analysis

For convergent validity, the AVE of each factor was estimated. The results showed that all the factors have values above.50, except for Passive Dropout Ideation, suggesting that this factor may require further refinement to improve its convergent validity.

Verification of the normality assumption indicated that the data does not follow a normal distribution, with a value of p < .001 for all variables. Consequently, Spearman’s rho was used to assess the correlations. As shown in Table 8, the correlations between the factors of the university dropout intention formation process instrument and the Willpower subscale were mostly significant, but of low magnitude, with only one reaching a moderate level. These results support the divergent validity of the instruments, suggesting that the dropout intention phases and willpower constructs assess distinct psychological dimensions. Further evidence of divergent or discriminant validity can be seen in Table 8. Here, the square root of the AVE for each construct is greater than its correlation with the Willpower scale. This supports the idea that the constructs are conceptually distinct [67].

**Table 8:** Convergent and Divergent Validity.

Variable	1	2	3	4	5	6	AVE	√AVE
**(1) Social Satisfaction**	–						0.555	0.745
**(2) Career Satisfaction**	.434***	–					0.543	0.737
**(3) Performance Satisfaction**	.439***	.526***	–				0.606	0.778
**(4) Passive dropout ideation**	–.263***	–.347***	–.359***	–			0.411	0.641
**(5) Active dropout ideation**	–.238***	–.325***	–.327***	.592***	–		0.673	0.820
**(6) Dropout Intention**	–.221***	–.340***	–.284***	.505***	.632***	–	0.728	0.853
**(7) Willpower**	.234***	.269***	.279***	–.140***	–.100**	–.136***	–	–

***Correlation is significant at the 0.001 level (2-tailed). **Correlation is significant at the 0.01 level (2-tailed).

## Discussion

The general objective of the present study was to develop and validate a multidimensional self-report instrument to assess the process of university student’s dropout intention formation as it evolves over their first-year experience. This study was fueled by the lack of adequate tools to do so and the need to capture a process with high impact on the decision to drop university studies. The main findings, implications, conclusions and limitations of this study are presented below.

Based on the three studies conducted, a theoretical model and an instrument composed of factors that allows to account for the development of university dropout intention formation process during the first year of university studies were developed. This instrument grounded and validated theoretically and empirically demonstrates a solid alignment between theoretical concepts and empirical measurements.

The results of the first study showed that the formation of dropout intention is a process involving reflections and internal evaluations regarding the desire to leave university. These thoughts emerge from the student’s perception of whether their social, academic, and performance expectations are being met-factors that are essential for adapting to the primary demands of the higher education context.

This process is structured by means of three interconnected phases that function dynamically: (Phase 1) Perception of satisfaction and adaptation; (Phase 2) Ideation of dropout; and (Phase 3) Dropout intention (see Fig 2). This novel perspective of dropout intention will allow for the assessment of intention to dropout and the possibility to implement improved strategies to reinforce retention intention in university students at risk, which is a contemporary priority [19,48,70].

The results of the second study helped improve the selection and improvement of items, demonstrating the relevance of content analysis in the design of new instruments and the value of the cognitive interview as a strategy to evaluate the items’ clarity, and the format and acceptability of the instrument by the target participants.

Finally, the results of the third study helped formulate an instrument to measure the Dropout Intention Formation Process, which is composed of 29 items distributed in 6 factors of first order that are organized in three phases (see Fig 4). The first corresponds to the phase of academic strategic adaptation, which entails social satisfaction, career satisfaction, and academic performance satisfaction. The findings on this study have the capacity to contribute to the understanding of the impact of social, career, and performance satisfaction on the emergence of university dropout intention formation. Recent research underscores the crucial role of friendships and social connections in university academic achievement and university retention intention [19,30] with socially connected first-year university students being more likely to be retained in their second year [71]. On the other hand, positive career satisfaction along with self-regulation [21] have an impact on the permanence intention. Procrastination leads to dissatisfaction, which drives students’ dropout intentions within first three years of study [23].

The next phase corresponds to the reflective phase in which previous experiences of dissatisfaction generate ideas, reflections and evaluations towards the desire to dropout from the university. In this phase, a distinction between passive ideation related to unsystematic and scattered ideas regarding the desire to drop out is made; it contemplates the possibility of dropping out without taking concrete or deliberate actions towards that end. Active ideation refers to frequent, systematic and structured thoughts of dropping out. It involves weighing the advantages and disadvantages of such a decision, as well as the impact of dropping out. The last phase corresponds to the pre-actional phase, related to thoughts that explicitly allude to dropping out higher education. The study provides relevant strong theoretically and empirically demonstrating that the phases of the instrument are highly related.

This research also showed that university dropout is not a sudden, impulsive or isolated decision, but rather a gradual process. As students accumulate experiences, they strengthen their decisions to retain their studies or to dropout. This gradual and procedural perspective conceives the intention to dropout as an intrapsychic construction -internal, subjective construction- [36,48], which is configured from significant experiences that occur in interpsychological spaces.

The instrument showed adequate reliability indices in most dimensions, assessed using various indicators, with the exception of the “Passive Dropout Ideation” dimension, which presented a relatively lower reliability (ω = .680; CR = .627); although still acceptable in the early stages of instrument development [72]. For example, Nunnally and Bernstein [73] suggest that reliability coefficients above 0.60 can be tolerated in exploratory research, while DeVellis [72] emphasizes that slightly lower alpha values could reflect a broader coverage of the construct, rather than low reliability. Overall, the results support the internal consistency of the instrument and its usefulness in evaluating the dimensions involved in the instrument.

During the EFA, some items showed cross-loadings. To ensure the clarity and parsimony of the factorial model, we followed the guidelines proposed by Howard [74], known as the.40–.30–.20 rule. According to this rule, an item is considered adequate if: (1) it presents a primary loading equal to or greater than.40; (2) it maintains secondary loadings below.30; and (3) the difference between the primary loading and the highest cross-loading is at least.20. Those items that did not meet these criteria were carefully evaluated for their possible impact on factorial validity. In line with this recommendation, we observed that most items maintained their secondary loadings below the.30 threshold, a commonly accepted criterion for considering that there is no problematic cross-loading. However, items 13 and 22 did not meet the minimum difference of.20 between primary and secondary loadings, and item 28 showed a cross-loading of.310, just above the threshold. Despite these observations, it was decided to retain these items in the model due to their high primary loading on the theoretically expected factor and their conceptual relevance within the instrument’s structure. This decision is based on psychometric practice, which recommends prioritizing the theoretical coherence of the model over the automatic elimination of items with marginal cross-loadings [65]. Overall, the instrument’s factorial structure is considered sufficiently clean, and each factor retains its original conceptual definition. However, it is suggested that future research seeking to validate the instrument in other samples or educational contexts consider reevaluating these items and exploring possible improvements in their factorial performance.

## Implications

These results have psychometric, educational and theoretical implications. From the psychometric point of view, this study enriches number of instruments available to measure university dropout. Furthermore, the UDIFP-29 offers a tool to assess the formation, offering a solid basis for future research and practical applications in the educational context. This instrument makes it possible to identify how the dropping out ideation is developing, so that it is possible to monitor and implement possible interventions to modulate the students experience so that the dropout ideation and subsequent dropout intention, are prevented.

In terms of educational implications, this instrument can facilitate the early detection of students at risk of dropping out and the implementation of prevention strategies, since it allows finding in which phase of the intention to dropout their career the students are. This permits institutions to tailor interventions more precisely according to the student’s current stage, improving the effectiveness of prevention strategies. For example, academic advisors can use the instrument’s results to provide targeted counseling focused on addressing the particular concerns or challenges faced by students in each phase, whether they relate to motivation, integration, or academic difficulties.

Moreover, retention programs can be designed or adjusted based on the profiles identified through the instrument, enabling proactive support such as mentoring, peer support groups, or skill-building workshops aimed at enhancing student engagement and persistence. As empirical evidence indicates [51], it´s imperative to have effective instruments for dropout intention detection in order to generate early interventions.

It is also important to note that this instrument was developed based on data from first-year university students, which is a period with high dropout rates [75]. This focus is particularly relevant because early university experiences often shape students’ long-term engagement and graduation success. By targeting this critical stage, the instrument provides higher education administrators, counselors, and educators with a contextually sensitive tool to monitor and support students when they are most at risk, thus strengthening institutional efforts to improve permanence and student outcomes.

This work has important theoretical implications for the understanding of dropout intention, taking into account that it is one of the few papers in which both a bottom-up and top-down bidirectional design process were followed. This approach incorporates complementary sources of validity and reliability, ensuring theoretical and empirical consistency. This approach reinforces the soundness of a theoretical model that underpins the instrument developed, evidencing its integrity and applicability.

### Limitations and future research suggestions

A key limitation concerns the sampling strategy: participants were selected through convenience sampling from a single higher education institution in Chile, predominantly representing students from the fields of engineering and health. This limited demographic and institutional scope restricts the generalizability of the findings, as the experiences and factors influencing dropout intention may vary significantly across different academic disciplines, universities, and cultural contexts. Future research should therefore aim to apply and validate the instrument across a more diverse and representative sample, including students from various regions, educational institutions, and fields of study both within Chile and internationally. Moreover, the sample size presented potential limitations for the robustness of the factorial analyses conducted. The division of the sample for exploratory and confirmatory factor analyses might have resulted in insufficient power according to some psychometric recommendations, which could affect the stability and replicability of the factorial structure.

Another limitation is that neither the predictive nor criterion validity of the instrument was assessed. There was no access to objective external measures, such as actual dropout data or observable behaviors related to dropout intention. This limitation prevents a more certain determination of the predictive power of the dimensions assessed on future academic behavior. It is recommended that future research use longitudinal designs and consider the inclusion of external indicators to strengthen the assessment of the instrument’s predictive validity. The longitudinal designs will allow to analyze how intention to dropout evolves over time, since dropout intention is a gradual process. In addition, it would be beneficial to develop additional instruments to measure different types of dropout intentions.

Despite these limitations, this research provides an instrument with sufficient evidence of validity and reliability, designed to be used in the context of higher education to effectively measure the process of formation of the intention to dropout in university students.

## Supporting information

S1 Appendix Instrument to evaluate the university dropout intention process. (DOCX)
