# Supplementary material for: Construction and validation of an instrument to assess the university dropout intention formation process UDIFP-29
Source: PLoS One. 2026 Jun 17;21(6):e0349293. doi: 10.1371/journal.pone.0349293 (PMC13274899; doi:10.1371/journal.pone.0349293)
Supplement: S1 Appendix — (DOCX) [file pone.0349293.s001.docx]

# S1 Appendix. Instrument to evaluate the university dropout intention process.

**Instructions: Using the following response scale indicate the degree of agreement with each of the statements presented:**

| **1** | **2** | **3** | **4** | **5** | **6** | **7** |
| --- | --- | --- | --- | --- | --- | --- |
| Strongly Disagree | Disagree | Slightly disagree | Neither agree nor disagree | Slightly agree | Agree | Totally agree |
| Totalmente en desacuerdo | En desacuerdo | Ligeramente en desacuerdo | Ni de acuerdo ni en desacuerdo | Ligeramente de acuerdo | De acuerdo | Totalmente de acuerdo |

| 1 | I have close personal relationships in the career. (Tengo relaciones personales cercanas en la carrera) | 1 | 2 | 3 | 4 | 5 | 6 | 7 |
| --- | --- | --- | --- | --- | --- | --- | --- | --- |
| 2 | I am adapted to the social environment of the career. (Estoy adaptado/a al ambiente social de la carrera) | 1 | 2 | 3 | 4 | 5 | 6 | 7 |
| 3 | I feel that I integrate with my classmates. (Siento que me integro con mis compañeros/as de clase) | 1 | 2 | 3 | 4 | 5 | 6 | 7 |
| 4 | I have good relationships with my classmates. (Tengo buenas relaciones con mis compañeros/as de clase) | 1 | 2 | 3 | 4 | 5 | 6 | 7 |
| 5 | I am satisfied with my social adjustment in my career. (Estoy satisfecho/a con mi adaptación social en la carrera) | 1 | 2 | 3 | 4 | 5 | 6 | 7 |
| 6 | I am satisfied with the number of subjects I have. (Estoy satisfecho/a con el número de asignaturas que tengo) | 1 | 2 | 3 | 4 | 5 | 6 | 7 |
| 7 | I am satisfied with the quality of the subjects I have. (Estoy satisfecho/a con la calidad de las asignaturas que tengo) | 1 | 2 | 3 | 4 | 5 | 6 | 7 |
| 8 | I am satisfied with the teachers I have. (Estoy satisfecho/a con los/as profesores/as que tengo) | 1 | 2 | 3 | 4 | 5 | 6 | 7 |
| 9 | I am satisfied with the contents of the subjects I have. (Estoy satisfecho/a con los contenidos de las asignaturas que tengo) | 1 | 2 | 3 | 4 | 5 | 6 | 7 |
| 10 | I am satisfied with the outcome of the evaluations. (Estoy satisfecho/a con el resultado de las evaluaciones) | 1 | 2 | 3 | 4 | 5 | 6 | 7 |
| 11 | I am satisfied with the learning I am achieving. (Estoy satisfecho/a con los aprendizajes que estoy logrando) | 1 | 2 | 3 | 4 | 5 | 6 | 7 |
| 12 | I am satisfied with the way I am studying. (Estoy satisfecho/a con la manera en que estoy estudiando) | 1 | 2 | 3 | 4 | 5 | 6 | 7 |
| 13 | I am satisfied with the academic goals I set for myself (e.g., getting a high grade). (Estoy satisfecho/a con los objetivos académicos que me propongo, por ejemplo, obtener una calificación alta) | 1 | 2 | 3 | 4 | 5 | 6 | 7 |
| 14 | I am satisfied with my academic to-do list. (Estoy satisfecho/a con la lista de tareas académicas que realizo) | 1 | 2 | 3 | 4 | 5 | 6 | 7 |
| 15 | I am satisfied with the organization of the academic tasks I perform. (Estoy satisfecho/a con la organización de las tareas académicas que realizo) | 1 | 2 | 3 | 4 | 5 | 6 | 7 |
| 16 | I am satisfied with the learning goals I set for myself. (Estoy satisfecho/a con las metas de aprendizaje que me propongo) | 1 | 2 | 3 | 4 | 5 | 6 | 7 |
| 17 | I am satisfied with the time I spend studying. (Estoy satisfecho/a con el tiempo que dedico al estudio) | 1 | 2 | 3 | 4 | 5 | 6 | 7 |
| 18 | I have been wishing for something to happen that would free me from going to university and/or studying. (He deseado que pase algo que me libere de ir a la universidad y/o estudiar) | 1 | 2 | 3 | 4 | 5 | 6 | 7 |
| 19 | I have had the desire not to get up to avoid going to university. (He tenido deseos de no levantarme para evitar ir a la universidad) | 1 | 2 | 3 | 4 | 5 | 6 | 7 |
| 20 | In short, I would rather not be studying at the university. (En definitiva, preferiría no estar estudiando en la universidad) | 1 | 2 | 3 | 4 | 5 | 6 | 7 |
| 21 | I thought about what I could do instead of studying for my degree. (He pensado sobre lo que podría hacer en lugar de estudiar la carrera) | 1 | 2 | 3 | 4 | 5 | 6 | 7 |
| 22 | I have been undecided about whether to continue studying this career. (Me he sentido indeciso/a sobre si seguir estudiando esta carrera) | 1 | 2 | 3 | 4 | 5 | 6 | 7 |
| 23 | I can't shake the feeling that I should give up my career. (No puedo deshacerme de la sensación de que debería abandonar mi carrera) | 1 | 2 | 3 | 4 | 5 | 6 | 7 |
| 24 | I am evaluating what it would mean for me to leave the career. (Estoy evaluando lo que significaría para mí dejar la carrera) | 1 | 2 | 3 | 4 | 5 | 6 | 7 |
| 25 | I am evaluating the advantages and disadvantages of abandoning my career. (Estoy evaluando las ventajas y desventajas de abandonar mi carrera) | 1 | 2 | 3 | 4 | 5 | 6 | 7 |
| 26 | I am reflecting on the short- and long-term consequences of leaving my career. (Estoy reflexionando respecto a las consecuencias a corto y largo plazo que tendría abandonar mi carrera) | 1 | 2 | 3 | 4 | 5 | 6 | 7 |
| 27 | I am thinking of dropout of higher education. (Estoy pensando abandonar los estudios superiores) | 1 | 2 | 3 | 4 | 5 | 6 | 7 |
| 28 | I am thinking of dropout of university for good. (Estoy pensando abandonar definitivamente la universidad) | 1 | 2 | 3 | 4 | 5 | 6 | 7 |
| 29 | I have thought about the possibility of dropout of higher education. (He pensado en la posibilidad de abandonar los estudios superiores) | 1 | 2 | 3 | 4 | 5 | 6 | 7 |
